# A new spanner in the works of bacterial transcription

**DOI:** 10.7554/eLife.02840

**Published:** 2014-04-22

**Authors:** Kristine B Arnvig, Finn Werner

**Affiliations:** 1 **Kristine B Arnvig** is in the Institute for Structural and Molecular Biology, University College London, London, United Kingdomk.arnvig@ucl.ac.uk; 2 **Finn Werner** is in the Institute for Structural and Molecular Biology, University College London, London, United Kingdomf.werner@ucl.ac.uk

**Keywords:** RNA polymerase, antibiotics, transcription, transcription initiation, inhibitor, bipartite inhibitor, *E. coli*

## Abstract

A promising molecular target that is unlikely to develop antibiotic resistance has been identified in bacteria.

**Related research article** Zhang Y, Degen D, Ho MX, Sineva E, Ebright KY, Ebright YW, Mekler V, Vahedian-Movahed H, Feng Y, Yin R, Tuske S, Irschik H, Jansen R, Maffioli S, Donadio S, Arnold E, Ebright RH. 2014. GE23077 binds to the RNA polymerase ‘i’ and ‘i+1’ sites and prevents the binding of initiating nucleotides. *eLife* **3**:e02450. doi: 10.7554/eLife.02450

**Image** The target site (green) of the antibiotic GE (blue) on RNA polymerase

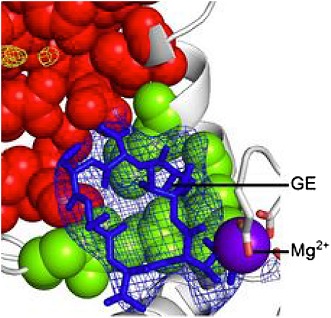

Bacterial infections cause over 17 million deaths globally every year (Butler and Buss, 2006; World Health Organisation, 2014). In the past 70 years, antibiotics have been critical in the fight against infectious disease, yet alarmingly, pathogenic bacteria of all categories have developed antibiotic resistance. This is rated one of the greatest threats to human health.

Alongside the rise of antibiotic resistant variants of already existing diseases, such as MDR-TB (multi-drug resistant tuberculosis) and MRSA (methicillin-resistant *Staphylococcus aureus*), new infectious diseases are emerging every year. However, few new antibiotics have been developed over the past two decades (Chief Medical Officer’s report, 2011). The situation is further complicated by the fact that many current antibiotics have severe side effects, particularly if they are used for a long time. There is, therefore, an urgent need for novel drugs. Now, in *eLife*, Richard Ebright and colleagues from Rutgers University, the Helmholtz Centre for Infection Research, and Naicons Srl—including Yu Zhang, David Degen, and Mary Ho as joint first authors—use a combination of genetic, biochemical and structural approaches to characterise how a recently discovered compound, with very promising properties, works (Zhang et al., 2014).

Designing drugs in a rational, systematic way requires an intimate knowledge of how they interact with their target and the mechanism by which they inhibit this target’s activity. Antibiotics act by interfering with processes that are essential to cells, such as transcription (Darst, 2004), translation or cell wall synthesis. In effect, these drugs ‘throw a spanner in the works’ of the bacterial cell. One set of prime targets for antibiotics are enzymes called multisubunit RNA polymerases (RNAPs), which transcribe the cellular genomes of all life on Earth (Werner and Grohmann, 2011).

The natural genetic mutations that constantly occur in all organisms mean that although a new antibiotic may initially successfully kill bacteria, it may not remain effective for long. Mutations that stop antibiotics inhibiting bacterial RNAP emerge randomly regardless of the environment the bacteria grow in. In most cases these changes will also make the bacterium less fit. Therefore, if the bacteria are not exposed to antibiotics, the number of resistant bacteria remains low compared to the number that are sensitive to the antibiotic (Comas et al., 2012). However, if antibiotics are then targeted at these bacteria, the selective pressure of the drug treatment may increase the proportion of drug-resistant mutants in the overall population, and thus a drug resistant strain of bacteria will emerge over time. In addition, the initial loss of fitness associated with the drug-resistant mutations is often compensated for by additional mutations, and so a new and more dangerous pathogen can evolve (Comas et al., 2012).

The ideal molecular target of an antibiotic—its binding site—would present little opportunity for drug resistance to evolve. Therefore, the target should be small, and directly involved in how RNAP works as an enzyme, to reduce the likelihood of a drug resistant RNAP evolving that remains catalytically active.

The bacterium *Actinomadura sp.*, which was originally isolated from a soil sample, produces an antimicrobial chemical called GE23077, henceforth referred to as GE (Ciciliato et al., 2004). GE is *Actinomadura*’s weapon in the fight for resources in its natural environment, and is used against fungi as well as Gram-negative and Gram-positive bacteria. Early studies demonstrated that GE was a potent inhibitor of RNAP purified from *Escherichia coli*. However, the compound was less effective against living cells as it is unable to cross the cellular envelope that surrounds a bacterium (Sarubbi et al., 2004). Improving GE’s properties, for example by increasing its uptake into a cell while not lowering its potency, requires the molecular basis of its activity to be thoroughly understood (Mariani et al., 2005).

RNAPs transcribe DNA by building long chains of RNA from individual nucleotides. GE, like another antibiotic called rifampin (Rif) that is used to treat tuberculosis, does not interfere with the first steps of transcription initiation. Therefore, RNAP can still find the correct start site and load the template DNA strand into its active site ready for RNA synthesis. GE inhibits the subsequent step, preventing the joining together of the first two nucleotide substrates in the RNA chain.

The X-ray crystal structures of bacterial RNAP in complex with GE solved by Ebright and colleagues provide a structural explanation for this activity (Figure 1). GE binding to RNAP interferes with the binding of the first and second RNA nucleotide substrates to the enzyme. Rif works in a similar way: as this antibiotic’s binding site is close to, but offset from GE’s, Rif allows the formation of di-and tri-nucleotides but prevents the synthesis of longer RNA chains. GE also interferes with the binding of a magnesium ion in the RNAP active site that is critical for enzyme catalysis.

**Figure 1.**
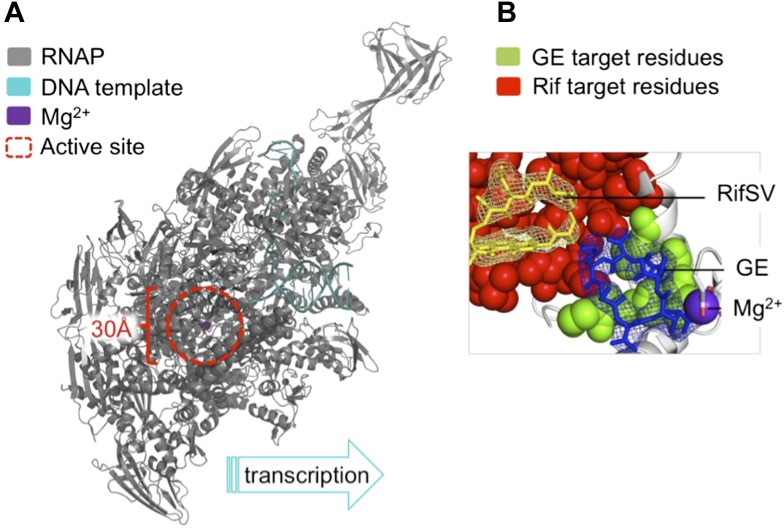
The antibiotic GE23077 (GE) targets a site on bacterial RNA polymerase to inhibit the initiation of DNA transcription. (**A**) The RNAP transcription initiation complex present in *Thermus thermophilus* bacteria. GE and Rif (as well as several other antibiotics) target the same region of the enzyme: the RNAP catalytic centre (highlighted with a red dashed circle). Zhang et al. found that the binding sites of GE and Rif are distinct, yet close to each other; this explains why the two drugs inhibit RNAP catalysis in subtly different ways. In both cases the antibiotic does not interfere with the first steps of transcription initiation, and the RNAP can still load a DNA template (teal). Instead, each antibiotic interferes with the next step: the bonding together of nucleotides to form an RNA chain. A saturation mutagenesis of a volume within 30 Å of the active site identified just 6 mutations that could give rise to GE antibiotic resistance, compared with 71 that produce Rif-resistance. (**B**) The RNAP active site. The GE (blue stick diagram) target site (green) identified by Zhang et al. is much smaller than Rif’s (yellow stick diagram) target site (red; image taken from Figure 6 of Zhang et al., 2014).

To assess the number of mutations at the binding site that confer GE resistance and thereby pose a threat to effective antibiotic treatment, Ebright and colleagues used a technique called saturation mutagenesis. This generates all the possible mutations at a specific site, in this case within 30 Å of the catalytic centre of *E. coli* RNAP. The analysis revealed a very small target-based resistance spectrum, as only a very few mutations could produce RNAPs that are both resistant to GE and still catalytically active. The GE target site is also roughly 10-fold smaller than that of Rif and is therefore superior as it presents fewer possible sites of mutation.

Since the GE and Rif binding sites are next to but distinct from each other, the two drugs do not display cross-resistance. Therefore, Rif resistant RNAPs are not resistant to GE, and vice versa. Moreover, GE and Rif can bind simultaneously to RNAPs. As the ‘icing on the cake’ of their work, Ebright and colleagues joined together GE and Rif to generate a novel two-part (bipartite) compound, Rifa-GE. While Rifa-GE is as potent as GE or Rif, it also effectively inhibits RNAP variants that are resistant to either.

Once GE-containing compounds have been modified so they are better able to enter a living bacterial cell, they are likely to become front-line drugs in the struggle against bacterial infections.
